# A Pictorial Review of the Many Faces of Gossypiboma – Observations in 6 Cases

**DOI:** 10.12659/PJR.900745

**Published:** 2017-08-01

**Authors:** Gayatri Pole, Bright Thomas

**Affiliations:** Department of Radiology, Luton and Dunstable University Hospital, Luton, U.K.

**Keywords:** Foreign Bodies, Gossypium, Surgical Sponges

## Abstract

Gossypiboma or textiloma is the result of a foreign-body reaction to extraneous material, usually a surgical sponge that was accidentally retained within the body.

The diagnosis of a retained surgical sponge is often delayed due to its infrequent occurrence and protean appearances.

The purpose of this pictorial review is to define the common sonographic and CT features of gossypiboma. A retrospective review of sonographic and CT images of 6 surgically proven cases of retained surgical sponges was undertaken.

## Background

Gossypiboma or textiloma is the result of a foreign-body reaction to extraneous material, usually surgical sponge that was accidentally retained within the body. This is most often associated with emergency procedures, prolonged surgeries, unexpected changes in the course of operation, excessive haemorrhage, change of personnel during the operation or inexperienced and inadequate number of staff [1].

Radiologically, foreign bodies can imitate tumours or abscesses. The manifestations of gossypibomas are protean, and diagnosis is often difficult. Gossypibomas are associated with severe complications leading to high patient morbidity. Moreover, gossypibomas have significant medicolegal implications [2].

Gossypibomas are frequently diagnosed in the intraabdominal cavity. However, they can also be found in the chest [3,4], extremities [5], CNS [6] and breast. Presentation may vary from immediate postoperative period to several decades after surgery [7]. Rarely, it may mimic a tumour and lead to unwarranted invasive diagnostic procedures or extensive extirpative surgery, which may result in further complications [8,9].

## Pathological Features

Pathologically, gossypibomas can produce two types of reactions in the body. One is an aseptic fibrinous response that results in adhesions or encapsulation leading to granuloma formation. The other is of an exudative nature leading to abscess formation, which may be complicated by secondary bacterial infection and fistula formation. The latter usually presents during the early post-operative period.

## Imaging Features

Radiological characteristics of gossypibomas are variable.

Sonography: Ultrasound often demonstrates a well-delineated mass containing a whorl-like, hyperreflective echo with a hypoechoic rim and strong posterior acoustic shadowing. Ultrasound characteristics can be classified into two types, a cystic type and a solid type. The former presents as a cystic lesion with a wavy echogenic structure within. The solid type can appear as a complex mass containing hyper- and hypoechoic regions. The retained material itself usually causes acoustic shadowing on ultrasound, although it may be due to calcified areas in the gossypiboma or pockets of air.

CT scan: CT may show a low-density, heterogeneous mass with a thick peripheral rim. The presence of mottled, bubbly gas shadows should prompt the diagnosis. The spongiform pattern with gas bubbles [5] is often considered as the most characteristic CT appearance of gossypibomas. The mass may contain wavy, striped, high-density areas that represent the sponge itself. Although it may be difficult to identify the type of retained material, some features may suggest certain objects. Linear densities with a peculiar infolding/whorled configuration suggest a towel as the cause [4,10]. On the other hand sponges as well as gel foam tablets appear as low-attenuation masses with multiple gas bubbles [5,10]. Calcification of the wall may be identified on CT as a dense peripheral rind.

## Complications

Gossypibomas may give rise to various complications depending on the type of pathologic response. While fibrinous reaction results in encapsulation and localisation, an exudative reaction will lead to extrusion of the foreign material through a fistulous track, either externally to the skin or internally into the rectum, vagina, bladder, or intestinal lumen [11]. This can cause intestinal obstruction [12], malabsorption and gastrointestinal haemorrhage [11]. Of note here is a case of retained surgical sponge within the large bowel which was later expelled by defecation.

## Multimedia

### Case 1

**Figure 1:**
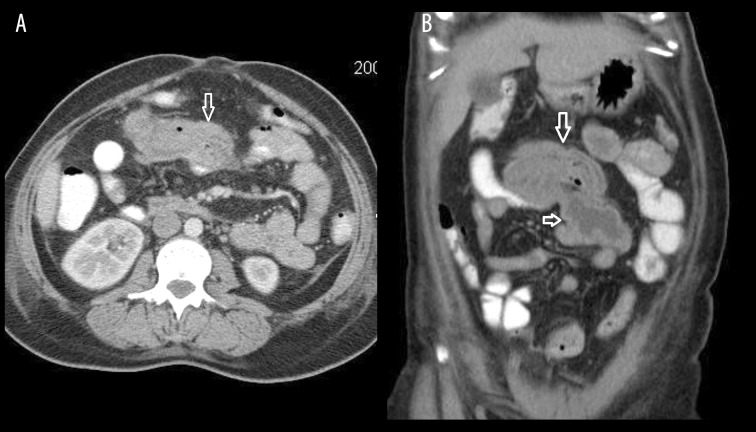
(**A**) Axial contrast-enhanced CT scan at the level of the upper pole of right kidney reveals inhomogeneous mass with gas bubbles within, caused by a retained sponge. (**B**) Coronal reformatted image shows the retained sponge, causing mass effect on an adjacent small bowel loop.

Abdominal gossypiboma following surgery for ruptured ectopic pregnancy. A 31-year-old female underwent surgery for ruptured ectopic pregnancy. One week later, she presented with vomiting and abdominal pain (Figure 1A, 1B).

### Case 2

**Figure 2:**
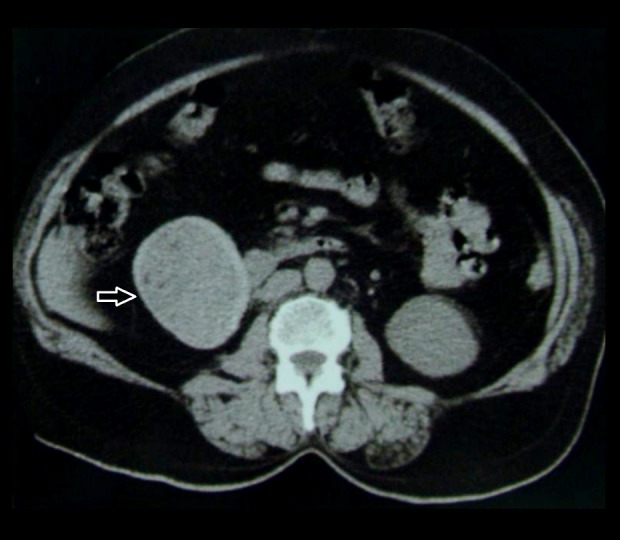
Enhanced CT scan shows a thick-walled structure with high-attenuation contents in the retroperitoneum on the right side.

A 55-year-old woman with retroperitoneal gossypiboma (Figure 2).

### Case 3

**Figure 3:**
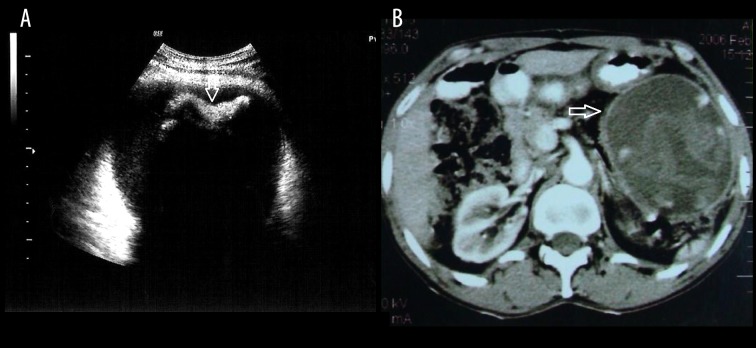
(**A**) Sonogram reveals a cystic lesion containing a linear, hyperechoic structure demonstrating dense posterior acoustic shadowing. (**B**) Contrast-enhanced CT scan of the abdomen shows a thin-walled, cystic mass in the left lumbar region with internal linear densities showing a characteristic infolding/whorled configuration.

A 45-year-old male with low-grade pain in the upper abdomen following gastrojejunostomy (Figure 3A, 3B).

### Case 4

**Figure 4:**
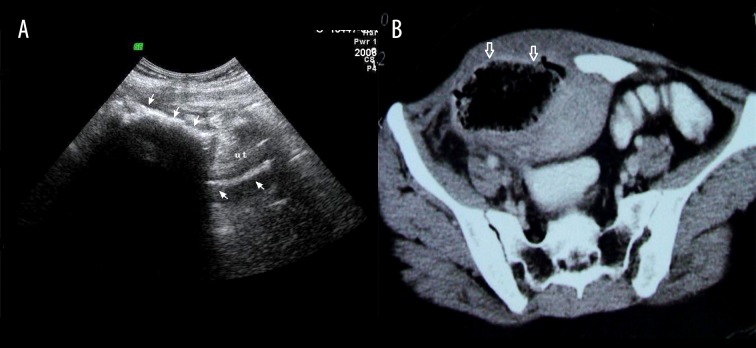
(**A**) Sonography shows a wavy, hyperechoic structure within the fundal region of the uterus. The endometrial echo of the lower uterine segment is well-delineated. (**B**) Contrast-enhanced CT at the level of the pelvis reveals a mass containing mottled, bubbly gas shadows protruding through the uterine fundus.

Intrauterine gossypiboma in a 23-year-old woman with pelvic pain and suppurative discharge 3 months after caesarean section (Figure 4A, 4B).

### Case 5

**Figure 5:**
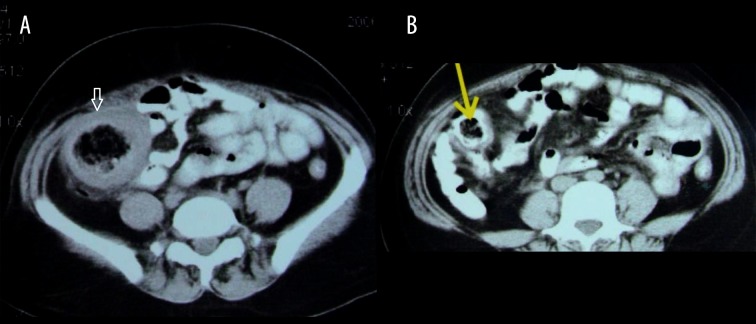
(**A**) Contrast-enhanced CT scan shows a thick-walled lesion in the right iliac fossa with central, mottled gas attenuation. (**B**) More caudal section reveals that the mass have migrated into the caecum.

Pelvic gossypiboma complicated by a fistulous connection with the caecum. A 40-year-old woman with pain in the right iliac fossa. The patient had a recent history of hysterectomy (Figure 5A, 5B).

### Case 6

**Figure 6:**
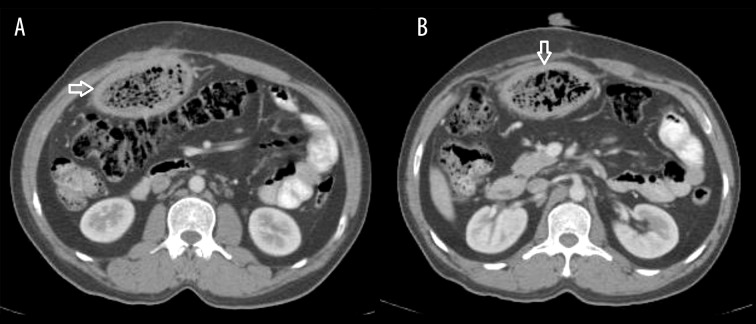
(**A, B**) Enhanced CT scan of the abdomen reveals a gas-containing, spongiform mass adherent to the anterior abdominal wall.

Intraabdominal gossypiboma in a 50-year-old woman with a history of prior surgery for gallbladder carcinoma who presented with abdominal pain and fever (Figure 6A, 6B).

## Conclusions

Gossypiboma is not an uncommon entity and the radiologist should be aware of its common and unusual appearances in order to make a correct diagnosis in the appropriate clinical setting.
